# Enzyme-accelerated CO_2_ capture and storage (CCS) using paper and pulp residues as co-sequestrating agents†

**DOI:** 10.1039/d3ra06927c

**Published:** 2024-02-20

**Authors:** Ayanne De Oliveira Maciel, Paul Christakopoulos, Ulrika Rova, Io Antonopoulou

**Affiliations:** a Biochemical Process Engineering, Division of Chemical Engineering, Department of Civil, Environmental and Natural Resources Engineering, Luleå University of Technology SE-97187 Luleå Sweden io.antonopoulou@ltu.se

## Abstract

In the present work, four CaCO_3_-rich solid residues from the pulp and paper industry (lime mud, green liquor sludge, electrostatic precipitator dust, and lime dregs) were assessed for their potential as co-sequestrating agents in carbon capture. Carbonic anhydrase (CA) was added to promote both CO_2_ hydration and residue mineral dissolution, offering an enhancement in CO_2_-capture yield under atmospheric (up to 4-fold) and industrial-gas mimic conditions (up to 2.2-fold). Geological CO_2_ storage using olivine as a reference material was employed in two stages: one involving mineral dissolution, with leaching of Mg^2+^ and SiO_2_ from olivine; and the second involving mineral carbonation, converting Mg^2+^ and bicarbonate to MgCO_3_ as a permanent storage form of CO_2_. The results showed an enhanced carbonation yield up to 6.9%, when CA was added in the prior CO_2_-capture step. The proposed route underlines the importance of the valorization of industrial residues toward achieving neutral, or even negative emissions in the case of bioenergy-based plants, without the need for energy-intensive compression and long-distance transport of the captured CO_2_. This is a proof of concept for an integrated strategy in which a biocatalyst is applied as a CO_2_-capture promoter while CO_2_ storage can be done near industrial sites with adequate geological characteristics.

## Abstract 2

Alternative routes for carbon capture and storage are needed. This article evaluates the carbonic anhydrase effect on improving CO_2_ capture with alkaline residues for application in mineral storage.

## Graphical abstract

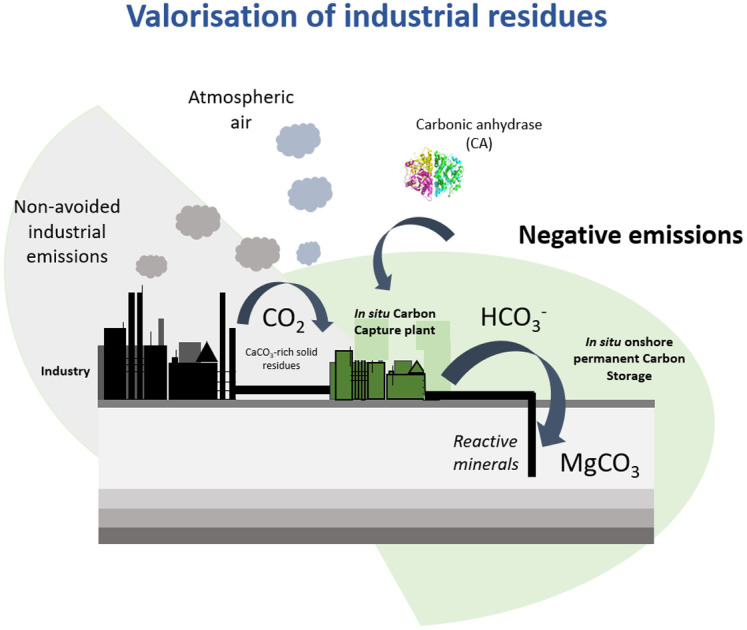

## Introduction

Carbon capture and storage (CCS) plays an important role in decreasing greenhouse gas (GHG) emissions and mitigating climate change. Industries, such as mining, cement, and pulp and paper, produce alkaline wastes, which have proven capabilities to work as CO_2_-sequestrating agents.^1–3^ Yearly, more than 7 billion tons of such residues are produced worldwide, representing a potential to capture and store 2.9–8.5 million tons of CO_2_ in the long term.^4^ Mineral weathering is a possible route to utilize alkaline residues in CO_2_ capture and can bring some benefits since it is a wet route, which can enhance the kinetics of the capture reaction without requiring high, or even any, additional power inputs.^5,6^ Carbonate rocks (such as limestone, CaCO_3_ and dolomite, CaMg(CO_3_)_2_) are abundant in nature and play an important role as natural carbon sinks, since they react with atmospheric CO_2_ (weathering). Nevertheless, the process happens in a geological timeframe, which is not quick enough for reaching the worldwide goals targeted for CCS by 2050.^7^ Consequently, carbon capture utilizing aqueous rich carbonate solutions and concentrated CO_2_ streams, such as industrial flue gases, has been gaining increasing attention as a path for accelerated weathering in the last decades.

Recently, a demonstration carbon-capture plant installed in a coal-fired power unit and using limestone as a co-sequestrant agent revealed a CO_2_-capture efficiency of up to 55%.^8^ The mechanism involved in calcium carbonate weathering (acidic conditions) can be explained by two distinct reactions, the first reaction being CO_2_ hydration (eqn (1)) and the second mineral dissolution (eqn (2)), of which the final targeted product is the formation of bicarbonates (HCO_3_). The carbonate equilibrium, which includes free CO_2_ or carbonic acid, bicarbonate ions, and carbonate ions, is highly pH dependent. At pH values <8.3, bicarbonates are predominant rather than carbonate ions. Alkali metal oxides are also relevant for CCS applications since in aqueous solutions these react with CO_2_ to also produce bicarbonates (eqn (3)). The weathering or mineral dissolution reaction can be favored at acidic conditions (pH < 7.5) for carbonates^9,10^ and oxides (pH < 8.4).^11–13^ The CO_2_ hydration reaction supplies the H^+^ protons for mineral dissolution and is the limiting step in the process of weathering since it has slow kinetics.

1 CO_2(g)_ + H_2_O ↔ HCO_3_^−^ + H^+^

2 MCO_3_ + H^+^ ↔ M^2+^ + HCO_3_^−^

3 M(OH)_2_ + 2H^+^ + 2CO_2(g)_ ↔ M^2+^ + 2HCO_3_^−^

where M is a metal, such as Ca or Mg.

Carbonic anhydrase (CA) is a metalloenzyme that catalyzes the limiting step in mineral weathering (CO_2_ hydration reaction (eqn (1))). It is one of the fastest existing enzymes with a turnover rate up to 10^7^ s^−1^, and has demonstrated potential for enhancing the yield in carbon capture, utilization, and storage (CCUS) applications,^14–16^ and also in accelerating the weathering of carbonates.^17–19^ Therefore, CA utilization could bring about enhanced mineral dissolution and a higher productivity of bicarbonates, which could then be converted further into a thermodynamically stable form (*i.e.*, mineral carbonation).

Silicate-rich mineral rocks have been demonstrated to be able to store CO_2_ permanently, in particular, Mg-silicates, such as olivine ((Mg,Fe)_2_SiO_4_), since their dissolution/carbonation also might occur under mild temperature and pressure conditions. The direct use of bicarbonate solutions, obtained from a CO_2_-capture process step, in replacement of the injection of pressurized CO_2_ gas in the bedrock, could be an alternative to overcome some issues such as gas leakage from storage sites,^20^ thus enabling safer geological carbon storage.

In contact with solutions rich in bicarbonates, under neutral to acidic conditions, silicates react with the H^+^ proton, releasing a metallic ion, such as Mg^2+^ in the case of forsterite (Mg_2_SiO_4_). This step is referred to as mineral dissolution or leaching of the mineral rock during the CO_2_ storage (eqn (4)). The leached Mg^2+^ ions can react with (bi)carbonate, according to the pH of the aqueous solution, and then form magnesium carbonates (MgCO_3_), which is referred to as carbonation (eqn (5)). The formed solid carbonates serve as a permanent storage form of CO_2_.

4 M_2_SiO_4_ + 4H^+^ ↔ 2 M^2+^ + H_4_SiO_4_

5 M^2+^ + CO_3_^2−^ ↔ MCO_3(s)_

where M is a metal, such as Mg in the case of olivine, forsterite, *etc*.

**Fig. 1:**
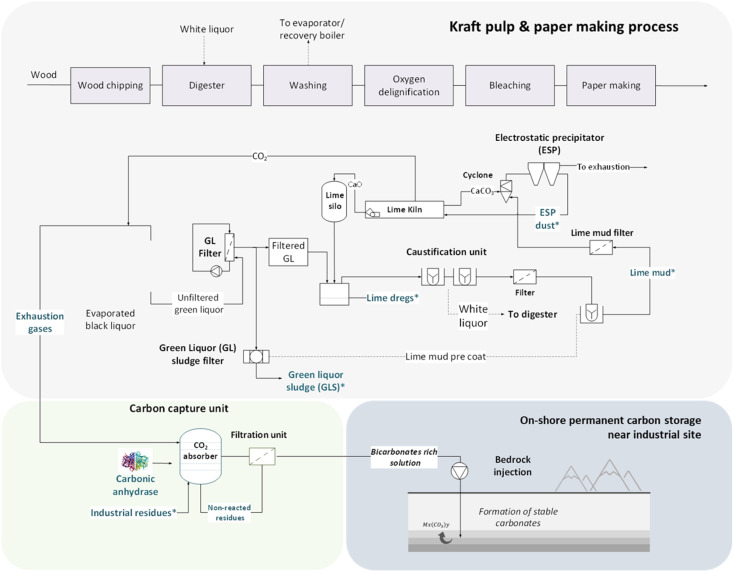
Proposed concept for an *in situ* CCS strategy utilizing solid residues from the pulp and paper industry.

Fig. 1 proposes a path to combine a process of carbon capture and storage for the pulp and paper industry. The synergies in respect with the logistics, energy efficiency, and the share of common industrial facilities could be a great asset to facilitate the development of CCS units in industrial sites. In Sweden alone, about 130 000 tons of waste generated from the pulp and paper sector was sent to landfills in 2019, which was subject to tax for waste generation (517 SEK per ton waste).^21^ Thus, the valorization of such residues could offer an alternative route for promotion of both CCS practices and the circular economy.

Aiming to integrate biotechnology to enhance CCS processes, this current study aimed to evaluate the application of industrial residues from the pulp and paper sector, composed majorly of CaCO_3_, as co-sequestrating agents for a CA-accelerated aqueous carbon-capture process. The utilized residues did not receive any prior treatment (acid, thermal, or mechanical activation^22^) to potentialize the carbon capture, which could imply lower costs. As a result of the CO_2_-capture step, a bicarbonate-rich solution was obtained by enzyme-accelerated CO_2_ hydration and mineral dissolution of the industrial solid residues, which was further used for mineral dissolution and the carbonation of olivine, a highly abundant mineral on earth, in order to understand its potential for the permanent storage of CO_2_.

For the expected outcomes, it would be desirable not only to accelerate the CO_2_-capture rate by use of an enzyme, but also to achieve the highest concentration of bicarbonates and the lowest pH value to further promote olivine dissolution and carbonation as a CO_2_-storage step. A CA mutant with high tolerance to alkaline environments was selected due to the nature of the tested waste. Research on CA application to boost both accelerated mineral weathering and increased bicarbonate production using alkaline wastes is still limited in the literature and has been tested only with well-known materials, such as limestone and brucite. Consequently, the proposed utilization of residues that might contain impurities, such as sulfates, phosphors, and metals, in CA-assisted CO_2_ capture will contribute to expanding the knowledge in this field.

## Materials and methods

### Enzyme production

The DNA sequence of an ultrastable CA mutant from *Desulfovibrio vulgari*s (DvCA8.0)^23,24^ was incorporated with 6xHis-tag and inserted between the NdeI and XhoI restriction sites of the pET22b(+) vector and transformed in *Escherichia coli* BL21. The pre-cultures were prepared using Luria Bertani medium containing 100 μg mL^−1^ ampicillin and incubated overnight at 37 °C. Cultures were done in an auto-induction medium containing lactose (ZYP-5052 without trace elements) and 100 μg mL^−1^ ampicillin and incubated overnight at 32 °C. The cells were harvested, resuspended in 100 mM pH 8.0 Tris–HCl buffer and lysed in a homogenizer at 700 bar. The produced lysate was filtered to 0.2 μm, freeze-dried, and resuspended in water to create a concentrated enzyme stock solution. Finally, the lysate was dialyzed in 20 mM pH 8.0 Tris–HCl buffer to minimize the buffer interference in the further reactions. Expressions were verified by a His-Tag purification step, SDS-PAGE, and by measuring the CA activity using standard assays (*p*-nitrophenyl acetate and Wilbur–Anderson assays) in lysates from transformed and non-transformed *E. coli* cultured cells.

### Characterization of the co-sequestrating agents

Four distinct industrial solid residues from the Kraft process – lime mud (R1), green liquor sludge (R2), electrostatic precipitator dust (R3), and lime dregs (R4) – were kindly provided by the paper and pulp industry firm Billerud (Karlsborg, Norrbotten, Sweden). X-Ray fluorescence (XRF, Hitachi) and X-ray diffraction (XRD, PANalytical) techniques were utilized for identifying the chemical composition and the phases of each material. The average particle size of the samples was estimated through a particle size and shape analyzer (Camsizer XT, Retsch Technology). The moisture content was determined using a moisture analyzer scale (Sartorius MA30). Due to the irregularity of R4, the material was ground in a roll mill to reach an average particle size <25 μm prior to the particle-size measurements. Scanning electron microscopy (SEM) analysis was conducted to observe the surfaces of the residues.

### CO_2_-capture trials

**Fig. 2:**
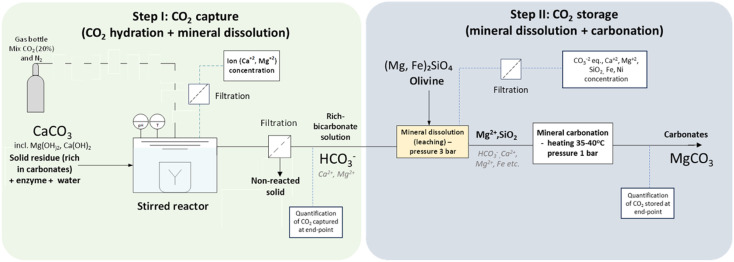
Experimental set-up for CCS in the laboratory. M: Metal.

Experiments were carried out in a 250 mL reactor at room temperature, using 167 mL working solution containing 0.4% w/w of industrial solid residue (DM basis) suspended in ultrapure water (18.2 MΩ cm^−1^). Next, 2.5 mL of concentrated enzyme lysate or 20 mM pH 8.0 Tris–HCl was added to the CA-added or control reactions, respectively. The DvCA8.0 concentration in the concentrated lysate was 27 mg mL^−1^ with a specific activity of 6690 WAU per mg. In the case of CO_2_ capture from the open air, the reactor used was a flat beaker left open to allow contact with atmospheric air. In the case of CO_2_ capture from synthetic gas (20% CO_2_ : 80% N_2_, Linde Gas, Luleå, Sweden) simulating industrial exhaust gases, a three-neck round-bottom flask was used instead. The gas was bubbled in the flask at a flow rate of 7.8 mL min^−1^. The reactors were agitated using a magnetic stirrer during the reaction. The pH value (pHenomenal, VWR, 1100L) and temperature were monitored over time, and at certain time intervals, aliquots were collected and filtered to 0.45 μm for determination of the Ca^2+^, Mg^2+^, and SO_4_^2−^ concentrations (Spectroquant ® test kits, Supelco). At the end of the reaction, the supernatant was filtered to 1.2 μm, and the amount of bicarbonate formed during the reaction was estimated from a modified biomineralization method.^25^ In short, a solution of 1.3 M Tris and 6.0% w/w CaCl_2_·2H_2_O was added to the filtered supernatant in a ratio of 1 : 4 v/v and agitated at 37 °C and 300 rpm for at least 12 h. The formed solid was filtered, washed, and then oven-dried at 60 °C for 24 h. The final weight of the dried formed matter was measured, and the material was subjected to XRD analysis. Quantification of the bicarbonates or total CO_2_ captured attributed to combined CO_2_ hydration (eqn (1)) and mineral dissolution (eqn (2) and (3)) was performed according to Section 1 of the ESI.† The unreacted industrial solid residues recovered by filtration were oven-dried at 50 °C and subjected to XRD analysis in order to identify the chemical composition and phases after the CO_2_-capture reaction. An overview of the CO_2_ capture set-up performed in the laboratory is presented in Fig. 2.

### CO_2_ storage using olivine as a reference material

Tests were performed according to the scheme process illustrated in Fig. 2. A solution rich in bicarbonate ions obtained in a CO_2_-capture step was recovered by filtration and utilized for mineral dissolution and carbonation. The supernatant was mixed with high purity milled and dried olivine (Ward's Sciences) at a concentration of 150 g L^−1^ in a 500 mL Duran bottle, pressurized at 3.0 bar with N_2,_ and left to react under stirring at 600 rpm and room temperature. Two systems were evaluated: one containing the supernatant from a CA-added CO_2_-capture reaction and one was the control, containing the supernatant from a CO_2_-capture reaction without CA. Every 24 h, an aliquot was withdrawn, filtered, and the concentrations of Ca^2+^, Mg^2+^, alkalinity (CO_3_^2−^/HCO_3_^−^), and SiO_2_ measured to monitor the dissolution of the olivine minerals over time (eqn (4)). The samples were also analyzed for Fe, Si, and Ni by ICP-SFMS according to SS-EN ISO 17294-2:2016 (ALS Scandinavia, Luleå, Sweden). After 5 days, the pressure was released from the bottle, and the bottle was sealed and then heated at 35–40 °C overnight to favor the carbonation of leached Mg^2+^ ions (eqn (5)). At the end of the reaction, the solid olivine residue was recovered through vacuum filtration, oven-dried, and kept for further analysis. Thermal gravimetric analysis (TGA; PerkinElmer TGA 8000; 10 °C min^−1^, 80 L min^−1^ N_2_) and XRD were performed on olivine, prior to and after mineral dissolution/carbonation, to identify the carbonates formed and the differences between the samples before and after the reaction, respectively. The quantification of the total CO_2_ stored was done according to Section 2 of the ESI.† The milled and dried olivine was characterized for its particle-size distribution by sieving and was then subjected to SEM analysis prior to its use in the CO_2_-storage experiments.

## Results and discussions

### Characterization of industrial solid residues

**Fig. 3:**
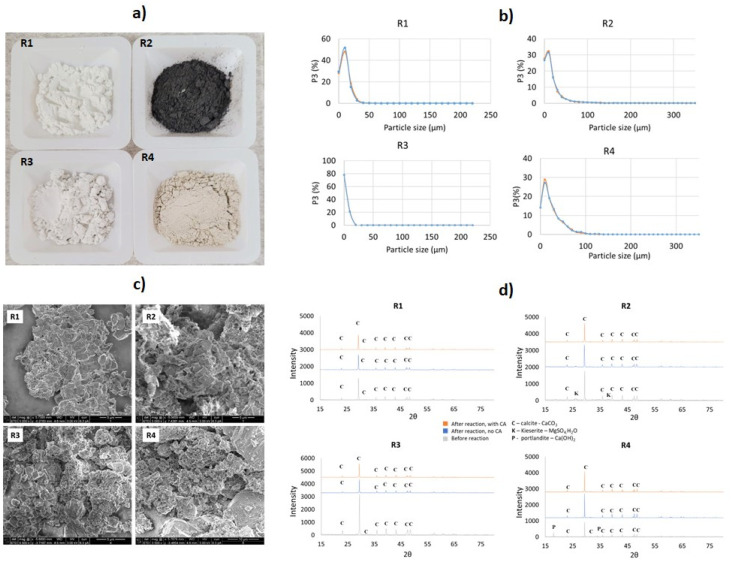
(a) Photos of the representative samples, (b) particle-size distributions, (c) SEM analysis and (d) XRD patterns of R1–R4 (R1: lime mud, R2: green liquor sludge, R3: electrostatic precipitator dust, and R4: lime dregs).

Except for R4, which was crushed and ground, all the residues were utilized as received. As shown in Table 1, the average particle size (*P*_size_) varied from 7.3 to 23.1 μm. The particle-size distribution is presented in Fig. 3b. The residues had moisture contents varying from 3.6% to 49% approximately, with R2 having the highest water content.

**Table 1:** Characterization of solid industrial residues from the paper and pulp industry (R1–R4)^a^

	Lime mud (R1)	Green liquor sludge (R2)	Electrostatic precipitator dust (R3)	Lime dregs (R4)
**Element composition (% w/w)**				
Mg	1.13 ± 0.09	4.96 ± 0.08	1.39 ± 0.03	1.56 ± 0.12
Al	0.10 ± 0.01	0.32 ± 0.01	0.10 ± 0.00	0.97 ± 0.04
Si	0.17 ± 0.01	0.60 ± 0.01	0.20 ± 0.02	0.54 ± 0.04
P	0.53 ± 0.01	0.17 ± 0.00	0.50 ± 0.00	0.30 ± 0.00
S	0	1.62 ± 0.00	0.14 ± 0.03	0.00 ± 0.00
K	0	0.40 ± 0.00	0.00 ± 0	0
Ca	38.42 ± 0.10	29.98 ± 0.10	37.74 ± 0.53	37.8 ± 0.86
Mn	0.03 ± 0.00	1.35 ± 0.02	<0.2	<0.2
Fe	0.03 ± 0.03	0.35 ± 0.01	<0.2	0.17 ± 0.00
Ni	<0.2	<0.1	<0.1	<0.2

**Particle size (μm)**				
*P*_size_ (μm)	13.99	16.75	7.3	23.1

**Chemical composition (% w/w)**				
CaCO_3_	96.1	75.0	94.0	90.8
CaO	N.D	N.D	N.D	2.1
MgSO_4_	N.D	6.1	N.D	N.D
MgO	N.D	8.1	N.D	N.D
Other phases	3.9 (metal oxides, alumino-silicates)	10.5 (alumino-silicates, metallic oxides, carbonides)	6.0 (silicates, oxides)	7.1 (silicates, metal oxides)

**Percentage dry matter (% w/w)**				
	83.4 ± 0.9	51.5 ± 1.4	96.4 ± 1.1	93.2 ± 2.4

a N.D: not determined.

Based on elemental analysis by XRF, all the analyzed materials presented a high concentration of Ca – varying from approximately 30.0% to 38.4% (Table 1). Other relevant elements identified were Mg, Al, Si, and S. Mn was found in significant concentrations (1.35%) for R2, as well as S (1.62%), indicating the possible presence of sulfates. Other elements, such as Cr and V, were found in smaller concentrations of <0.15% in all the materials. The XRD results (Fig. 3d) showed that CaCO_3_ was the majority phase in all the samples. For R2, the presence of MgO and Mg(SO_4_)_2_ was also detected. R4 presented peaks for Ca(OH)_2_ besides the presence of CaCO_3._ Despite the identification of Mg in all the residues by XRF, XRD analysis only indicated corresponding magnesium-containing phase peaks for sample R2, possibly because this material presented higher concentration than the others. Small amounts of CaO could be also expected in sample R3, since they shared parts of the same process (lime kiln), as shown in Fig. 1. Other materials, such as aluminum silicates or silica, appeared in smaller amounts; however, they were not detected during the XRD analysis. In order to estimate the concentrations of the existing phases, a mass balance was performed according to the methodology described in Section 3 of the ESI.†

Fig. 3a presents the distinct materials after drying and milling. SEM analysis indicated the presence of rhombohedral calcite in all the analyzed residues (Fig. 3c). The R1 and R3 samples had high homogeneity and contained mostly CaCO_3_. R2 showed the presence of flower-like structures, possibly due to the presence of Mg(OH)_2_. R4 had the most irregular structure and bigger grain size when compared to the other residues, indicating the presence of other phases, such as silicates and Ca(OH)_2_.

### CO_2_ capture from open-air reactions

#### Mineral dissolution (Ca^2+^, Mg^2+^)

**Fig. 4:**
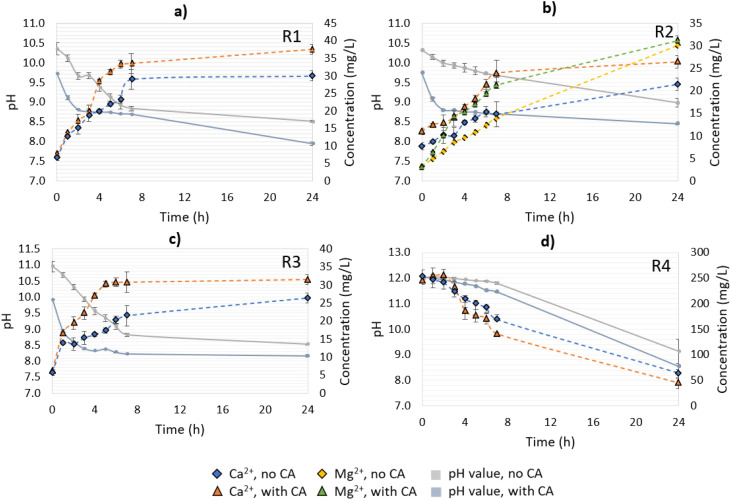
pH variation and ions (Ca^2+^, Mg^2+^) release over time during CA-accelerated and non-CA-accelerated CO_2_ capture in open-air reactions. (a) R1: lime mud, (b) R2: green liquor sludge, (c) R3: electrostatic precipitator dust, and (d) R4: lime dregs.

For residues R1, R2 and R3, the increase in Ca^2+^ concentration over time was correlated to the pH drop – due to the formation of H^+^ from the CO_2_ hydration step (Fig. 4a–c). The addition of CA boosted the mineral dissolution in terms of the absolute values for all the studied materials, and R1 exhibited the highest Ca^2+^-leaching reaction in the presence of the enzyme – 37.5 mg L^−1^ – followed by R3. An increment in Ca^2+^ concentration of up to 1.25-fold was observed in the CA-added reactions for R1, and 1.19-fold for R3. Sample R2, instead, exhibited a lower dissolution rate of Ca^2+^ over time, especially in the first 3 h of reaction, when compared to R1 and R3 (Fig. 4b). A possible explanation for this was the lower concentration of CaCO_3_ in R2 compared to the other materials, and the presence of Mg(OH)_2_, which would compete with the available CO_2_ for CaCO_3_ dissolution. When compared to the non-enzymatic reaction, the CA-added system showed a release of Mg^2+^ varying from 1.9–2.1-fold in the first 4 h, with a subsequent decline as the systems converged to a similar pH value (app. 8.5–9.0). Additionally, Mg^2+^ ions under alkaline conditions and at low concentrations (0.8 × 10^−3^ mol kg^−1^)^26^ were demonstrated to inhibit calcite dissolution – leading to a reduced Ca^2+^ concentration, as observed in this experiment.

Differently from the other materials, R4 had a higher Ca^2+^ concentration at time 0 of the reaction, which decreased over time (Fig. 4d). This can be explained because of the presence of Ca(OH)_2_, which will form CaCO_3_ when in contact with the available CO_2_, and precipitate in the solution. Since the solution pH was higher than 11 over the first 7 h of the CO_2_-capture experiment, the formation of carbonate ions predominantly occurred over bicarbonates, enabling the free Ca^2+^ to react with them, leading to precipitation. Between 7 < pH < 10, bicarbonate and carbonate ions co-existed, and the former were favored, leading to a great reduction in the CaCO_3_-precipitation rate.^27^ At the end of experiment (24 h), the no- and CA-added R4 reactions presented pH values between 8.6–9.1, which were slightly higher than the other residues, which varied from pH 8.0–8.5. Note that between 7 and 24 h, the system containing R4 had greater pH value variation (2.7–2.9) than the other materials (0.1–0.7), indicating that R4 might require more time to react with the CO_2_ until reaching a stable pH value. As a general observation, the addition of CA resulted in a faster drop in the pH value over time for all the materials in comparison to the no-CA-added reactions, demonstrating the enhancement effect of the enzyme in accelerating the CO_2_ capture, and in the formation of H^+^ ions, as has been already observed in previous studies using limestone and dolomite and bovine carbonic anhydrase (BCA) for accelerated CO_2_ capture.^19^

Other works involving the mineral dissolution of materials rich in calcium carbonate under atmospheric CO_2_ concentration proved that CA played an important role in increasing the Ca^2+^ release, ranging from 2.3–11.7-fold in comparison to their control samples.^17,18,28^ In contrast, the present study, performed under non-optimized conditions, presented an increase in Ca^2+^ leaching of up to 1.25-fold. As a possible improvement, the utilization of buffered solutions, a higher speed of agitation, and a longer time of reaction (for R4) could result in higher CO_2_ mass transfer to the solution, and consequently higher Ca^2+^ leaching and bicarbonates production. Also, the residues employed in our tests did not receive any treatment to minimize any existent impurities, such as organics and heavy metals, which can impair the enzyme activity and mineral dissolution. Nevertheless, CA presented a positive effect on the results, and since we aimed at the utilization of enriched CO_2_ streams (*i.e.*, industrial flue gases), reactions using gas with an enriched CO_2_ concentration were performed as well. More details about the pH value variation as well as the Ca^2+^ and/or Mg^2+^ concentrations for R1–R4 are presented in Fig. 4.

#### CO_2_ capture and bicarbonate quantification

**Fig. 5:**
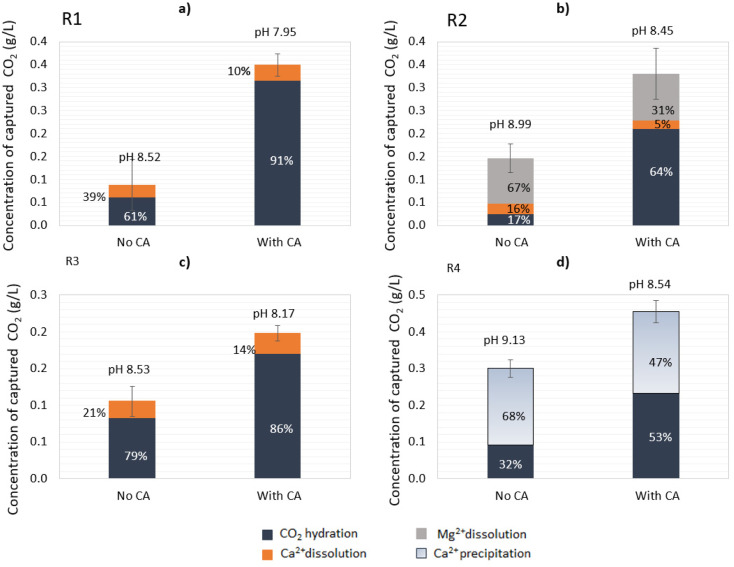
Quantification of the captured CO_2_ at the end of the open-air reactions (24 h). (a) R1: lime mud, (b) R2: green liquor sludge, (c) R3: electrostatic precipitator dust, and R4: (d) lime dregs.

From the quantification of the total bicarbonate formed in the solution in the CO_2_-capture reaction, the effect of the enzyme in the concentration of captured CO_2_ was calculated. For all cases, the addition of CA increased the CO_2_ hydration reaction extent and decreased mineral dissolution as a percentage of the total CO_2_ capture (Fig. 5). For instance, in the case of R1, addition of the enzyme increased the ratio of CO_2_ hydration over mineral dissolution from 6.1 : 3.9 to 9 : 1 (Fig. 5a). Under given conditions and based on the CA-accelerated reaction, the highest amount of total CO_2_ captured was achieved in descending order for: R4 > R1 > R2 > R3. In particular, the addition of CA in R1 increased the CO_2_ capture 4.0-fold, in R2 2.3-fold, in R3 1.8-fold, and in R4 1.5-fold, as determined at the end point (24 h). The maximum concentration of bicarbonates (HCO_3_^−^) measured in solution for R1 in the CA-accelerated reaction at the end point was 0.48 g L^−1^ (corresponding to 0.35 g L^−1^ captured CO_2_). For R4, 53% of the total amount of CO_2_ sequestrated was captured in the form of carbonates that were not present in the aqueous solution, and 47% was captured in the form of soluble bicarbonates (Fig. 5d). Thus, the concentration of bicarbonates available in solution after the end of the reaction for R4 in the CA-accelerated reaction was 0.32 g L^−1^ (corresponding to 0.23 g L^−1^ captured CO_2_).

Despite the Ca^2+^ release being lower in R2, the dissolution of Mg(OH)_2_ contributed to the total bicarbonate concentration in this system, reaching a similar concentration as observed with R1 (Fig. 5b). With R4, part of the CO_2_ from the capture was destined for the carbonation of Ca(OH)_2_, which was evident by the decrease in Ca^2+^ in the solution over time, so the final concentration of bicarbonate measured did not reflect the total amount of CO_2_ captured. There was an additional amount of captured CO_2_ that precipitated toward CaCO_3_, thus the total amount of CO_2_ captured could be attributed to CO_2_ hydration and Ca(OH)_2_ carbonation. Of course, there was also an ongoing reaction of mineral dissolution for CaCO_3_ (Ca^2+^ increase); however, this was not evident due to the predominant reaction of Ca(OH)_2_ carbonation (Ca^2+^ precipitation, decrease). Thus, it is important to underline that the attribution of the CO_2_-capture step to different reactions (hydration, dissolution, carbonation) was only apparent and depended on the components detected over time in the aqueous working solution.

Subsequently, notwithstanding the pH drop over time leading to mineral dissolution, the precipitation of carbonates could also occur in the opposite direction in small proportions for R1–R3, as the carbonation of CaCO_3_ (and MgCO_3_ for R2) is favored in neutral to alkaline pH values. Besides an improved CO_2_ capture, a high bicarbonate availability is essential in the mineral carbonation step, so both outcomes should be balanced when selecting which material has the highest potential to be applied in an industrial process.

### CO_2_ capture from CO_2_-rich gas

#### Mineral dissolution (Ca^2+^, Mg^2+^)

**Fig. 6:**
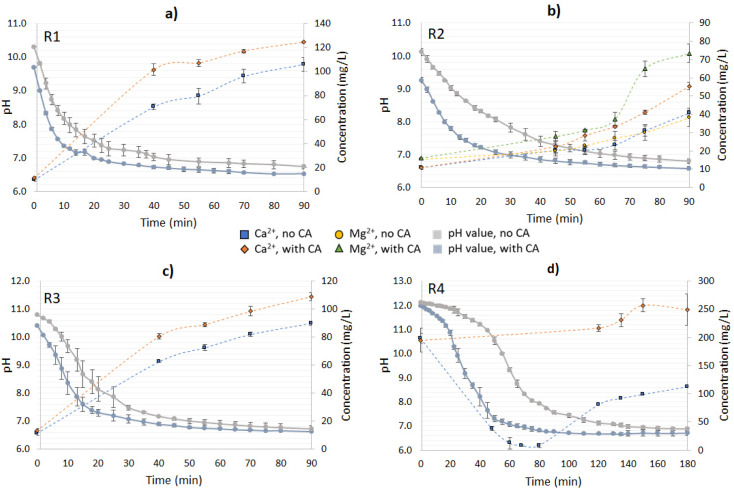
pH variation and ions (Ca^2+^, Mg^2+^) release over time during CA-accelerated and non-CA-accelerated CO_2_ capture from synthetic gas (20% CO_2_). (a) R1: lime mud, (b) R2: green liquor sludge, (c) R3: electrostatic precipitator dust, and (d) R4: lime dregs.

All tested solutions had pH values lower than 6.7 at the end of their reactions (90 min for R1–R3 and 180 min for R4); therefore a higher release of Ca^2+^ was observed when compared to in the open-air reactions (Fig. 4 and 6, respectively). For R1–R3, the collection of samples for analyzing the released ions concentration (Ca^2+^ and Mg^2+^) started when a pH value lower than 7.3–7.1 was reached (at 40 min for R1–R3 and at 120 min for R4), since the mineral dissolution benefits from neutral to acidic conditions. R1 presented the highest leaching of Ca^2+^, followed by R3 and R2. The final concentrations of Ca^2+^ measured in the CA-added tests with R1 and R3 (125 and 109 mg L^−1^, respectively) were similar to that found in a closed equilibrium system of CaCO_3_–H_2_O–CO_2_ near equilibrium conditions.^29^ The Mg^2+^ leaching in R2 was more affected by the presence of the enzyme than the Ca^2+^ release – by 1.9-fold and 1.4-fold compared to the non-enzyme added reactions, respectively. This was also observed in the open-air reaction but also was in accordance with another study that evidenced that brucite exhibited a dramatic dissolution rate growth in neutral to acidified environments (below pH 8.0).^12^

The CA-added system for R1 reached a pH value of 7.0 in 22.5 min of reaction; without the enzyme, instead, almost three times as much time was required to reach a similar condition (Fig. 6a). Since the reaction was performed until 90 min, the residence time of the non-CA-added system with R2 in acidic conditions was also reduced, leading to a lower dissolution of Mg(OH)_2_, as evidenced during the time interval between 65–75 min of the reaction. For the CA-added system, the pH value at 65 min was 6.68 while it was 6.99 for the no-CA-added system. The pH plays an important role in the dissolution of Mg(OH)_2_/MgO, and the two are exponentially related,^12^ which could explain the boost during the referred to timeframes. Also, the release of Ca^2+^ was considerably lower when compared to the other residues, possibly influenced by the Mg^2+^ inhibition effect on CaCO_3_, as also observed in the open-air reactions (Fig. 6b). The concentration of sulfates was measured for the systems added to R2; however, there was no significant variation of their concentration during the time of the reaction (data not shown).

The reactions with R3 revealed a similar trend to those with R1 in terms of Ca^2+^ release; however, they had a slightly higher pH value at time 0, possibly due to the presence of small contents of CaO, causing a “delay” in the pH drop in the first minutes of the reaction when compared to R1. Additionally, an improvement of 1.21-fold was verified in comparison to the no-CA-added reaction, at the time of 90 min (R3) (Fig. 6c). Similar results in the Ca^2+^ leaching enhancement were found in a system using bacterial CA and limestone, which exhibited a boost of 1.22–1.61-fold in comparison to the control reaction.^30^ In other works utilizing CA for accelerated weathering, the enzyme had a positive effect on the mineral dissolution, enhancing it in a range of 1.6–4.0-fold when compared to the control reactions.^28,31,32^

For R4, a longer time was required to give the system time to reach neutral to acidic conditions (pH below 7.3), as happened with the other materials (Fig. 6d). For the control reaction, a decrease in Ca^2+^ concentration was observed in the first 120 min of the reaction (pH > 7.3), which was attributed to Ca^2+^ precipitation in the carbonation of Ca(OH)_2_. The results indicated that when the solution reached pH 11 almost 80% of the dissolved Ca^2+^ ions had already precipitated, and the free Ca^2+^ comprised less than 8.0% of the initial concentration in the range of 10 < pH < 8. From 120 min (pH 7.3) until the end of the reaction at 180 min, Ca^2+^ started getting released again due to the mineral dissolution of CaCO_3_. Interestingly, in the CA-accelerated reaction, a decrease in Ca^2+^ concentration was not observed at all, as was observed for the respected open-air reaction. The Ca^2+^ concentration was instead rather stable and even increased slightly, indicating that there was an ongoing competitive reaction between Ca(OH)_2_ carbonation and mineral dissolution.

R4 and R2 presented the highest leaching improvements because of the presence of the enzyme (Ca^2+^ and/or Mg^2+^), despite R2 having a considerably lower absolute concentration of Ca^2+^ compared with the other materials. Therefore, it was also relevant to evaluate the rate of ions dissolution over time as a metric reaction development. With R1 and R3, the highest dissolution rates were observed at 40 min – meaning the dissolution rate had reached its peak at that time or before – and the addition of the enzyme increased their dissolution rate 1.47- and 1.31-fold compared to the no-CA-added reaction, respectively. For R2, the dissolution rate reached the peak enhancement at 55 min, corresponding to an 1.8-fold increase compared to the system without the enzyme. R4 had the highest dissolution rate at 150 min, with a CA-boosting effect of 2.2-fold in comparison with the non-enzyme assisted reaction. Table 2 displays the Ca^2+^ dissolution rate results for the distinct materials tested.

**Table 2:** Ca^2+^ dissolution rate of industrial solid residues (R1–R4) with and without the addition of CA

Dissolution rate ([ion] per min)								
Time (min)	Lime mud (R1)		Green liquor sludge (R2)		Electrostatic precipitator (R3)		Lime dregs (R4)	
	No CA	With CA	No CA	With CA	No CA	With CA	No CA	With CA
40	1.53 ± 0.21	2.26 ± 0.15	1.27 ± 0.11	1.68 ± 0.0	—	—	—	—
45	—	—	—	—	0.21 ± 0.02	0.25 ± 0.01	—	—
50	1.27 ± 0.13	1.74 ± 0.11	1.11 ± 0.13	1.38 ± 0.03	0.17 ± 0.01	0.21 ± 0.02	—	—
65	—	—	—	—	0.19 ± 0.02	0.35 ± 0.01	—	—
70	1.24 ± 0.15	1.51 ± 0.07	1.01 ± 0.09	1.22 ± 0.04	—	—	—	—
75	—	—	—	—	0.27 ± 0.02	0.40 ± 0.03	—	—
90	1.07 ± 0.08	1.26 ± 0.10	0.87 ± 0.10	1.06 ± 0.02	0.34 ± 0.02	0.49 ± 0.02	—	—
135	—	—	—	—	—	—	0.73 ± 0.03	0.93 ± 0.06
150	—	—	—	—	—	—	0.61 ± 0.05	1.34 ± 0.02
180	—	—	—	—	—	—	0.53 ± 0.03	0.55 ± 0.04

Industrial residues rich in oxides^33^ and hydroxides^34–37^ have high CO_2_ affinity and have proven their potential for carbon capture;^38,39^ however, materials rich in carbonates without previous treatment (*i.e.*, calcination, acidification) have been not widely exploited in the literature in direct carbonation studies, since they are not as reactive as their decarbonated forms. CO_2_ hydration is known to benefit from alkaline pH values, so the accelerated weathering of carbonates (also alkaline) is also an alternative for CO_2_ capture, without requiring extreme process conditions (high temperature and pressure). In this present work, utilizing residues as received from the industry and working under ambient temperature and pressure conditions, the CO_2_ capture was improved in the presence of CA, confirming the potential of this biotechnological route for application in carbon capture. Table 3 summarize studies on accelerated weathering with and without CA.

Mineral weathering, with and without CA^f^

**Table:** 

Without CA									
Co-sequestrating agent	Concentration (g L^−1^)	Temperature (°C)	Pressure (bar)	Volume of reaction	CO_2_ intake	Released ions concentration (mg L^−1^)	CO_2_ uptake	Reaction time	Ref.
Steel slag – Ca–K silicates	33.3	25 and 90	Ambient pressure	0.2 L	Saturated CO_2_ solution with injection 30 mL min^−1^	∼105–200 mg L^−1^ (Ca^2+^)	N.Q.	240 h	39
Red mud – NaOH	100	Room temperature	Ambient pressure	0.2 L	15.0%, 5 mL min^−1^	330 mg L^−1^ (Na^+^) and 173 mg L^−1^ (Al)	N.Q.	24 h	37
Coal fly ashes – alkaline oxides	100–200	Room temperature	Ambient pressure	0.5–1.0 L	Atmospheric	503–1073 mg L^−1^ (Ca^2+^)	N.Q.	24 h	40
Calcinated oil shale limestone – CaO rich	10–100	Up to 950	Ambient pressure	∼0.25 L	16%, 150 mL min^−1^	∼2820 mg L^−1^ (Ca^2+^)	N.Q.	1 h	41
Brucite – Mg(OH)_2_	7	Room temperature	15 atm	0.3 L	100% CO_2_, 15 atm	14.7–17.2 g L^−1^ (Mg^2+^)	65.9–71.0 g L^−1^	2.25 h	42
Limestone – CaCO_3_	5	Room temperature	1.5 bar	5 columns of 0.16 m^3^–30 m^3^ h^−1^ (continuous flow)	∼10% CO_2_, 50–200 m^3^ h^−1^	N.Q.	11 mM^a^	—	8

a Value of total alkalinity.

b Considered as the maximum Mg^2+^ concentration increase before nucleation – baseline 4.11 mg L^−1^.

c Based on dissolved inorganic carbon (DIC) concentration – includes both carbonates and bicarbonates.

d Total concentration of bicarbonates measured – includes the dissolution of the minerals.

e Values include measurements from the supernatant and mycelia.

f N.Q.: not quantified.

**Table:** 

With CA										
Co-sequestrating agent	Concentration (g L^−1^)	Enzyme	Temperature (°C)	Pressure (bar)	Reactor size	CO_2_ intake	Max released ions concentration/CA enhancement	CO_2_ uptake	Reaction time	Ref.
Brucite ore – Mg(OH)_2_	50	Bovine CA (BCA)	Room temperature	Ambient pressure	0.3 L	10%, 54–2700 mL min^−1^	∼3.7 mg L^−1^^b^	12.5 mg C per L^c^	3–11 days	34
Wollastonite – CaSiO_4_	2.3	CA from *Bacillus mucilaginosus*	35	Ambient pressure	0.05 L	Atmospheric and 3.9% CO_2_	∼25–66 (Ca^2+^), 1.19–1.22-fold	N.Q.	8 h	31
Limestone and dolomite – CaCO_3_ and CaMgCO_3_	—	CA from *Bacillus cereus*	Room temperature	Ambient pressure	30 × 3.0 cm diameter – 3 mL min^−1^ continuous flow	0.035–100%, 190 mL min^−1^	∼55–250 (Ca^2+^), 1.62–2.44-fold	∼160–850 mg L^−1^^d^	25 min	30
Wollastonite – CaSiO_4_	10	CA from *Aspergillus nidulans*, whole cells	37	Ambient pressure	0.1 L	Atmospheric and 3% CO_2_	Up to 550 mg L^−1^^e^, up to 18-fold	N.Q.	2–10 days	43
Limestone – CaCO_3_	5	Extracellular CA from *Aspergillus penicillium*, whole cells	28	Ambient pressure	0.4 L	Atmospheric	Up to ∼135 mg L^−1^, ∼2.3-fold	N.Q.	72 h	18
Limestone – CaCO_3_	0.2	Extracellular CA from *Bacillus* sp. GLRT102Ca	Room temperature	Ambient pressure	0.4 L	Atmospheric	9.7 mg L^−1^, 2.4–11.7-fold	N.Q.	9 h	17
Limestone – CaCO_3_	10	CA from *Bacillus mucilaginosus* – lysate and whole cells	32	Ambient pressure	0.01 L	Atmospheric	Up to 1130 mg L^−1^–4.0–6.0-fold	N.Q.	6 days	28
Pulp and paper residues – CaCO_3_ and alkaline oxides	4.0	Evolved CA from *Desulfovibrio vulgaris* (DvCA8.0)	Room temperature	Ambient pressure	0.25 L	20% CO_2_, 7.8 mL min^−1^	Up to 132 (Ca^2+^) and 62 (Mg^2+^), 1.2–2.2-fold	0.84–1.05 g HCO_3_^−^ per L or 0.15–0.18 g CO_2_ per g residue^a^	1.5–3 h	This study (not optimized)
Pulp and paper residues – CaCO_3_ and alkaline oxides	4.0	Evolved CA from *Desulfovibrio vulgaris* (DvCA8.0)	Room temperature	Ambient pressure	0.25 L	Atmospheric	Up to 35 mg L^−1^ (Ca^2+^), 2.2–3.1-fold	0.28–0.48 g HCO_3_^−^ per L or 0.024–0.042 g CO_2_ per g residue^a^	24 h	This study (not optimized)

#### CO_2_ capture and bicarbonate quantification

Under the given conditions and based on the CA-accelerated reaction, the highest concentration of formed bicarbonates was achieved in descending order for R1 ≥ R4 > R3 > R2. In particular, the addition of CA increased the CO_2_ capture as quantified in the first 40 min of reaction 2.2-fold for R1, 1.5-fold for R2, 1.5-fold for R3, and 1.6-fold R4. The maximum concentration of bicarbonates (HCO_3_^−^) measured was app. 1.0 g L^−1^ for R1 (corresponding to 0.74 g L^−1^ captured CO_2_), similar to the results found in a study utilizing bacterial CA (*Bacillus cereus* GRLT202) and limestone, also rich in CaCO_3_.^30^ This amount was almost 3 times higher than that in the respective open-air reaction and achieved in 1/16th of the time, resulting in an almost 50 times higher productivity.

**Fig. 7:**
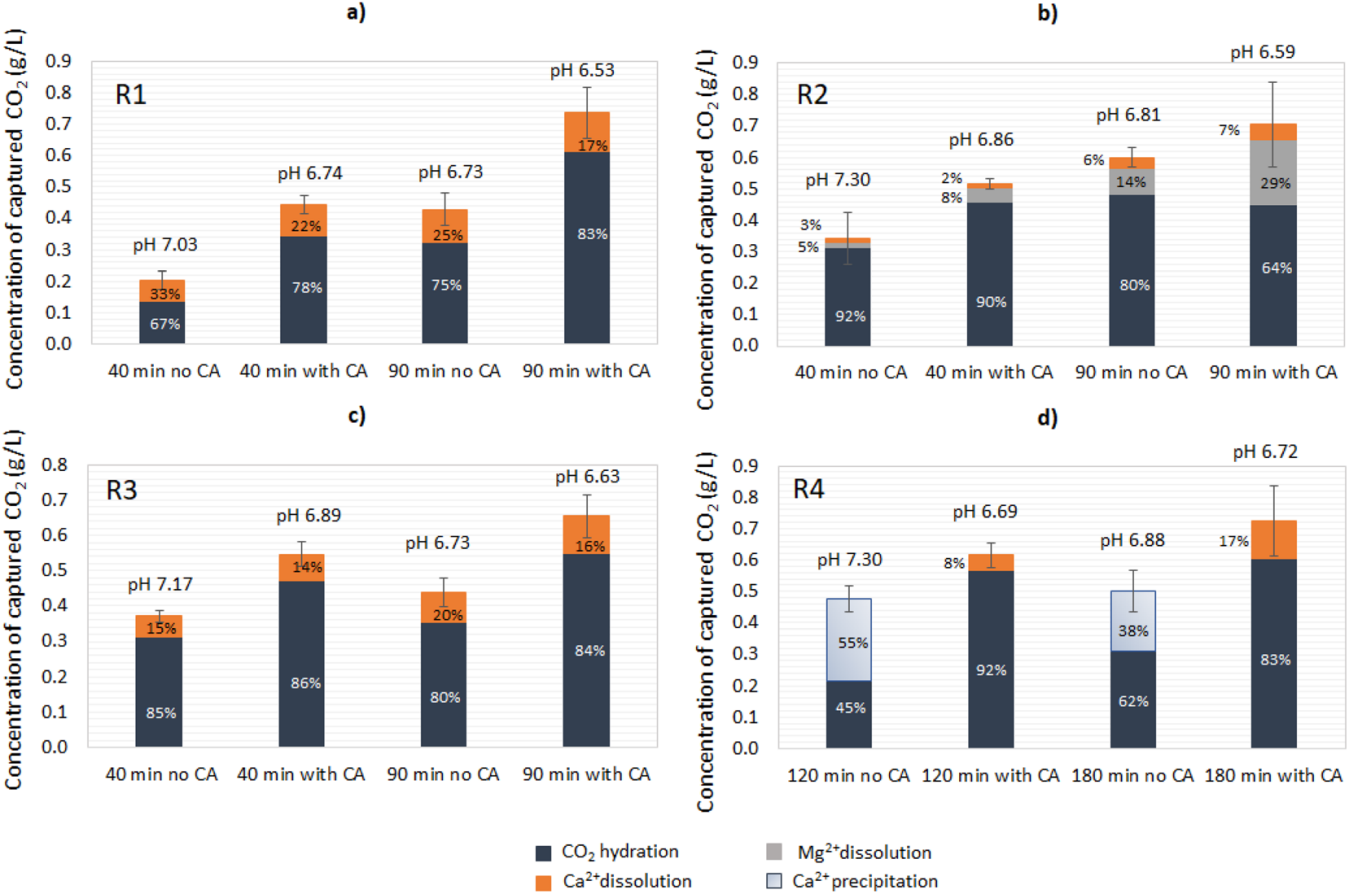
Quantification of CO_2_ captured at 40 and 90 min (end of the reaction) when synthetic gas (20% CO_2_) was used as the feedstock: (a) R1: lime mud, (b) R2: green liquor sludge, (c) R3: electrostatic precipitator dust, and (d) R4: lime dregs.

During the first part of the reaction (until the pH reached ∼7.3), the bicarbonate formation had contributions both from the CO_2_ hydration reaction and the mineral dissolution. As the reaction approached its end (90 min for R1–R3 and 180 min for R4), the CaCO_3_ and Mg(OH)_2_ dissolution rates were reduced, and CO_2_ hydration played a bigger role in the bicarbonate formation. Longer reaction times could also boost the mineral dissolution, however, at slower rates. Overall, the enzyme presence increased the yield of CO_2_ captured for all the materials. In contrast to the open-air reactions, the addition of CA had a varying response in regards to the extent of the CO_2_ hydration ratio over mineral dissolution as a percentage of the total CO_2_ capture. For R1 and R3, the addition of CA did not significantly increase the ratio of CO_2_ hydration over mineral dissolution neither at 40 nor at 90 min (Fig. 7a and c). For R2, the addition of CA did not significantly affect the ratio of CO_2_ hydration at 40 min and at 90 min, however the percentage of dissolved Mg(OH)_2_ over the total CO_2_ capture increased (Fig. 7b). Interestingly, at 90 min, the addition of CA led to a decrease in the CO_2_ hydration extent from 80% to 64% and a significant increase in the Mg(OH)_2_ dissolution, from 14% to 29%. For R4, the control reaction showed predominant Ca^2+^ precipitation, as in the open-air reactions. Interestingly, the addition of CA significantly accelerated the reaction, leading to apparent Ca^2+^ dissolution (Fig. 7d). This implies that there was ongoing competing Ca(OH)_2_ carbonation and CaCO_3_ dissolution, where CaCO_3_ dissolution was more predominant.

Notwithstanding the positive obtained results, the yield of CO_2_ capture and bicarbonate production could be optimized. It is important to emphasize that the current study was carried out at room temperature and pressure and the conditions were not optimized; thus, there is a window of opportunity for future improvements that could positively influence the CO_2_ capture and storage reactions. Increasing the partial CO_2_ pressure could lead to an enhancement in Ca^2+^ and Mg^2+^ leaching and a higher concentration of final bicarbonates. The temperature effect also needs to be analyzed since it promotes the kinetics of mineral dissolution, although high temperatures also decrease CO_2_ solubility, and could lead the bicarbonate ions to decompose. Finally, a higher concentration of co-sequestrant materials and increased CO_2_ flow rate are to be tested in further studies.

#### Characterization of the non-reacted residues

The solid residues (R1–R4) were characterized by XRD analysis before and after reaction to observe the phase variation in the residues. For R2 and R4, a clear difference was observed in the peaks corresponding to Ca(OH)_2_ and Mg(OH)_2_ (ESI†), which were absent after the CO_2_ capture reaction, possibly meaning they had reacted for form carbonates (CaCO_3_) and bicarbonates, respectively, as discussed in previous sections.

Also, no other CaCO_3_ crystalline forms were identified than calcite in all the analyzed samples, which could imply that the carbonation of Ca(OH)_2_ led to the same crystalline phases already present in the sample before the reaction or to amorphous/not detected CaCO_3_ phases. MgCO_3_ formation was not detected in the non-reacted solid from R2, excluding the presence of a ‘competitive’ Mg^2+^ precipitation reaction during CO_2_ capture.

### CO_2_ storage using olivine as a reference material

#### Olivine characterization

**Fig. 8:**
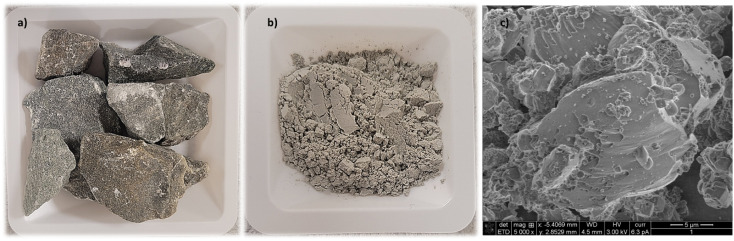
(a) Uncrushed olivine; (b) olivine crushed to a powder; (c) SEM analysis of the crushed olivine.

As expected, the XRF results demonstrated that there was a predominance of the elements Mg, Si, and Fe in the olivine sample (Table 4). The measured moisture content was up to 3.0% before drying. Ca, Cr, Mn; Ni, V, and Al were also detected at concentrations lower than 1.0%. From the XRD analysis, it was identified that the major phase of the sample were forsterite–fayalite, followed by calcium carbonate, and clinochlore at smaller proportions (Table 5). Metal elements, such as Mn and Ni, can commonly substitute in the olivine crystalline structure,^44^ explaining their presence in the XRF results. The received sample to be utilized in the carbonation experiments was crushed to powder to an average size of 20 μm < *P*_size_ < 37 μm. Fig. 8a and b show the olivine sample as received and after being crushed to a powder.

**Table 4:** Element composition of olivine^a^

Element	Mg	Si	Fe	Ca	Cr	Mn	Al	Ni	V
Composition (% w/w)	24.01 ± 0.19	18.92 ± 0.03	7.65 ± 0.02	0.98 ± 0.01	0.62 ± 0.01	0.12 ± 0.01	0.42 ± 0.02	0.335 ± 0.02	0.200 ± 0.001

a Reported values are for elements with concentration >0.1%.

**Table 5:** Chemical composition of olivine

Chemical formula	(Mg_1.57_Fe_0.21_)SiO_4_	CaCO_3_	(Mg,Al)_6_(Si,Al)_4_O_10_(OH)_8_	Other phases
Name of the mineral	Forsterite	Calcium carbonate	Clinochlore	Silicates and oxides
Composition (% w/w)	91.2	2.6	1.3	4.9

#### Olivine dissolution (Mg^2+^, Si)

**Fig. 9:**
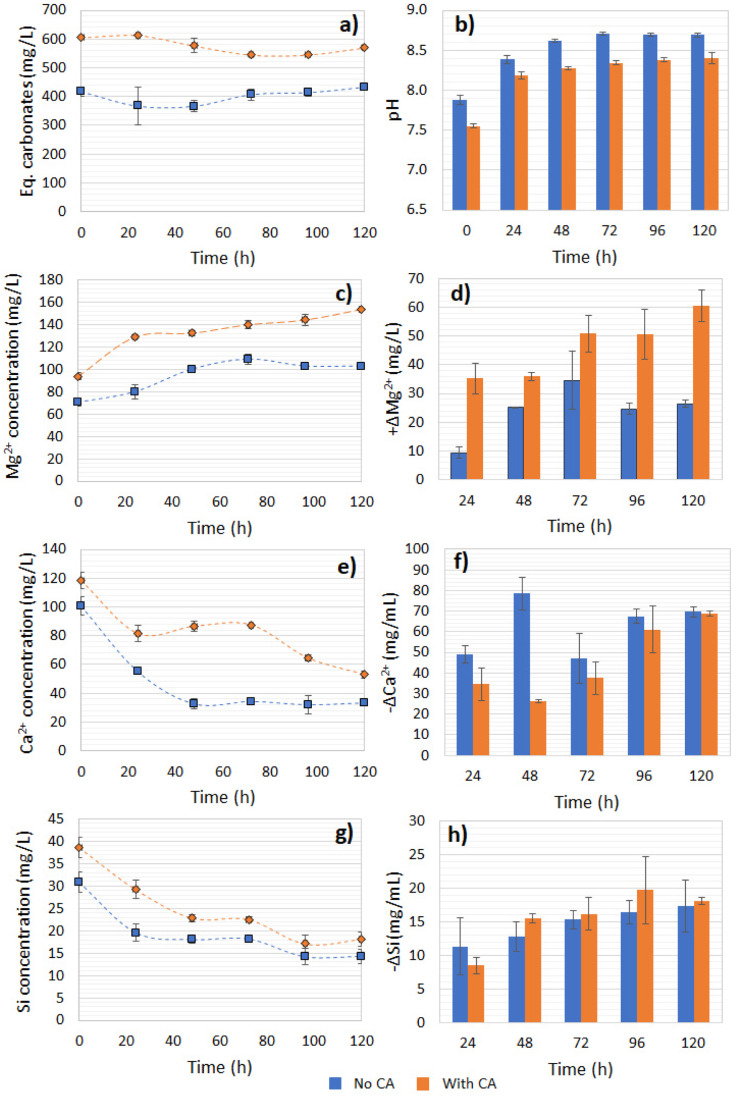
(a) Captured CO_2_ concentration, (b) pH, (c) Mg^2+^ concentration, (d) Mg^2+^ increase, (e) Ca^2+^ concentration, (f) Ca^2+^ decrease, (g) Si concentration, and (h) Si decrease over time during olivine dissolution. Δ*M* is defined as *M*_*t*_ − *M*_*t* = 0_.

The olivine powder was reacted with the rich bicarbonate solutions obtained from CO_2_ capture (using R1, with and without CA). The alkalinity, pH, and Ca^2+^, Mg^2+^, and Si concentrations were monitored for 120 h, as presented in Fig. 9. Over the 120 h of the experiment, the total alkalinity of the supernatant with bicarbonate without CA did not change while bicarbonate with CA presented a small decline (up to 5.7%), indicating that almost all the dissolved carbonate equivalents (both carbonates and bicarbonates) remained in the solution (Fig. 9a). Although the bicarbonate solutions had a slightly acidic pH value (6.5–6.7) after the end of the CO_2_-capture step, the moment the solution was mixed with the insoluble olivine at *t* = 0 min, the pH increased to 7.5–7.9 (Fig. 9b). During the time of the dissolution reaction, the pH increased and stabilized to more alkaline values of 8.5–8.7. In the preliminary experiments, when olivine was mixed with ultrapure water, an instant initial release of Mg^2+^ (22.5 mg L^−1^) was observed (data not shown). On the contrary, during olivine dissolution with bicarbonate, the Mg^2+^ concentration detected at *t* = 0 min, was 76.4 mg L^−1^ for the no-CA-added bicarbonate, and 93.1 mg mL^−1^ for the CA-added bicarbonate. This means that the actual Mg^2+^ concentration of the leached mineral and that due to the presence of H^+^ at *t* = 0 min was 53.9 mg mL^−1^ and 70.7 mg L^−1^, respectively.

In the reaction with bicarbonate formed from R1 and CA, the measured release of Mg^2+^ was higher compared to the control after 120 h of reaction, which was expected since the solution had a higher availability of H^+^, thus favoring dissolution of the olivine (Fig. 9c). At the end of 120 h, the Mg^2+^ ions release was 1.49-fold higher for the CA-added system compared to the no-CA-added one (Fig. 9d). The initial Mg^2+^ and Ca^2+^ concentration in the supernatant obtained from the reaction with the enzyme was approximately 20% higher than the one without CA, as observed at time = 0 of the dissolution reaction. Interestingly, the Ca^2+^ concentration dropped over time in both systems, indicating the precipitation of Ca^2+^. The available CO_2_ concentration was mostly stable over time in the solution (Fig. 9a). As suggested in a previous study, olivine dissolution leads to the formation of orthosilicic acid and other polymeric silica forms, which can potentially adsorb Ca^2+^*via* SiOH bonds.^45^ This phenomenon could be a possible explanation for the Ca^2+^ concentration decrease in our study, as it could be attributed to Ca^2+^ bonding onto the silicates surface (Fig. 9e) or the formation of other identified salts, such as CaSiO_4_. Nevertheless its formation did not seem to be likely since the reaction conditions, at slightly alkaline and with the presence of HCO_3_^−^, should have a contrary effect on the CaSiO_4_ precipitation rate.^46^ Besides, the Ca^2+^ concentration decline was more accentuated in the reaction using bicarbonate solution without the use of CA in the CO_2_-capture reaction (Fig. 9f). Such a reaction appeared to be fast in both systems, as the consumption of Ca^2+^ reached a plateau and stabilized after only 48 h. The Si concentration in the solution decreased over time in both systems, reaching a plateau at 48 h, and presented a decreasing concentration of soluble Si corresponding to 0.76–0.20 of the expected stoichiometry, indicating its precipitation to other insoluble phases (such as SiO_2(s)_) (Fig. 9g and h), and possible interactions with Ca^2+^ ions, reducing its concentration over time. Another case is that Ca^2+^ ions could have precipitated to CaCO_3_, which could have benefited from the higher pH values. Based on the carbonate concentration data (Fig. 9a), some CaCO_3_ could be formed in the CA-added system, since the total alkalinity concentration decreased by 5.7% at the end of 120 h. However, based on the stoichiometry, if all carbonate had reacted to form CaCO_3_, this could account for a maximum of only 68% of the total Ca^2+^ consumption. Except for Ni, which presented a concentration up to 31.7–74.1 μg L^−1^, the samples were tested for other elements at the ppm level, but no other mineral traces were found in the aqueous phase. Despite Fe accounting for approximately 7.7% of the total mass content of the olivine, an Fe concentration <0.02 mg L^−1^ was detected at the end of the mineral dissolution (120 h), evidencing that the forsterite (Mg_2_SiO_4_) underwent predominant dissolution when compared to the fayalite (FeSiO_4_) and agreeing with previous studies that indicated that preferential Fe dissolution benefits from acidic environments instead of alkaline conditions.^47^

A correlation between a lower pH value, dissolved CO_2_ (bicarbonate), and ions leaching was expected. In the bicarbonate solutions from CA-accelerated and non-CA CO_2_ capture and after mixing with olivine powder, the initial pH values were 7.55 and 7.88, respectively, with a higher alkalinity (bicarbonates dissolved) measured for the first system, which promoted higher Mg leaching. Other studies utilizing metal alkali silicates have confirmed a similar trend. In a batch CO_2_ pressurized system (2–10 atm), utilizing a pulp of serpentine at 150 g L^−1^, 19.4–32.7% leaching of the total magnesium of the mineral was recorded,^48^ demonstrating the higher the pCO_2,_ the higher the HCO_3_^−^, thus the greater leaching of Mg^2+^. Similarly, a positive correlation between the HCO_3_^−^ concentration and the release of Ca^2+^ ions was demonstrated in a system containing Wollastonite – a Ca-silicate mineral (CaSiO_3_) – in a near neutral pH range of 7–8.^46^

According to our results, the leached Mg represented only 0.4% of the total amount contained in the olivine sample. The supernatant's bicarbonate concentration was estimated to be not more than 0.02 M, which could impact the dissolution of the forsterite due to the limitation of H^+^ protons available. Besides, a favored leaching of Mg^2+^ was demonstrated to occur under acidic to slightly alkaline conditions (pH < 8). As proposed in other studies, the forsterite dissolution rate could be controlled by breaking the layers of the polymerized Si dimer precursor^47^ (>Si_2_OH^+^), whereby H^+^ protons would be exchanged by Mg^2+^ ions, and we hypothesized that a similar behavior could be observed in our experiments As the pH of the system increased, a different dissolution mechanism dominated, which could be highly inhibited by the presence of carbonate ions.^49^ Agreeing with this, the highest Mg leaching was observed in the first 24 h for both the no- and CA-added systems, when their pH values were the lowest, meaning the dissolution rate could be affected as the pH increased over the progress of the experiment. Besides, the formation of Si-rich layers in the mineral could impede the H^+^ from reaching the surface of the solid, impairing its dissolution.

In another example study that performed the aqueous carbonation of magnesium silicates, a Mg leaching of 17.3% was found.^50^ Nevertheless, the experiments were conducted under multiple cycles of absorption and with a higher CO_2_ partial pressure, which facilitated the dissolution of the mineral. Also, temperature plays a role in the kinetics of forsterite dissolution, as demonstrated previously under ambient pressure conditions and acidic conditions.^51^ In the present study, the average temperature for conducting the experiments was kept between 20–22 °C. As a suggestion for improvement in future works, tests with more concentrated bicarbonate solutions and high temperature should be included.

#### Mineral carbonation (MgCO_3_ formation)

**Fig. 10:**
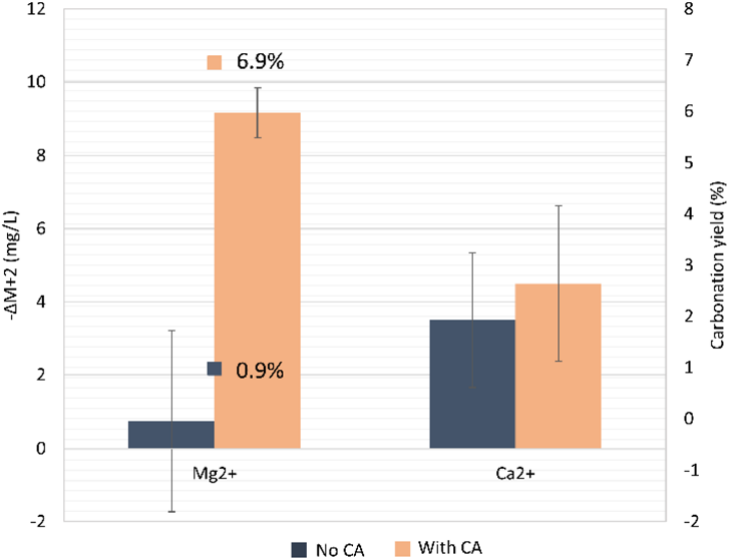
Carbonation yield for the conversion of Mg^2+^ ions in the formation of MgCO_3_, at the end of the mineral carbonation. The decrease in concentration of Mg^2+^ and Ca^2+^ during the carbonation step is also shown. Δ*M* is defined as *M*_*t*_ − *M*_*t*=0_.

Directly after the mineral dissolution step and to perform the carbonation step, the bottle was depressurized and heated to 35–40 °C overnight. The supernatant of the mixture contained mainly bicarbonate, silicate, Mg^2+^, Ca^2+^, Fe, and Ni. In this step, both Mg^2+^ and Ca^2+^ ions could possibly be converted to carbonates. However, the Ca^2+^ ions, which were carried along in the supernatant from the CO_2_ capture step and mostly did not derive from olivine, were consumed to a great extent during the prior step of olivine dissolution, probably due to their adsorption onto silica's forms and their reaction to produce CaCO_3_.^52^ Also, Mg^2+^ exhibited a lower preference than Ca^2+^ in the carbonation reaction, since it had a higher hydration energy than the latter, which is the limiting step in magnesium carbonates precipitation, making their precipitation unlikely at room temperature, as observed during the step of mineral dissolution.^53,54^ Indeed, during the olivine carbonation experiments, the Ca^2+^ concentration decreased only slightly (only by 5–7 mg mL^−1^) since most ions had reacted already (Fig. 10). Moreover, the lower pH value and higher concentration of Mg^2+^ (potential inhibitor^55^) could explain the undetectable yield from potential Ca^2+^ carbonation. Thus, only during the previous step of olivine dissolution did the Ca^2+^ ions react predominantly with the dissolved anions as soon as they were mixed with the olivine powder, since there was a pH shift from acidic (pH ∼ 6.6) to slightly basic conditions (>7.5), favoring their precipitation (Fig. 9b). The results revealed that the system without the presence of the enzyme had a higher precipitation (71.3%) of Ca^2+^ compared to the CA-added system (53.9%) during the dissolution step (Fig. 9e).

The targeted product in the olivine carbonation step in order to confirm the CCS concept is the formation of MgCO_3_. To quantify the carbonation yield, we looked into the decrease in available Mg^2+^ in the solution during the carbonation step. As a starting point, we considered the end of olivine dissolution step. In the reaction where bicarbonate from a CA-accelerated CO_2_ capture step was used, the precipitation into carbonates was 7.1-fold higher when compared to the no-CA-added system. Possibly, the higher concentration of bicarbonates and Mg^2+^ helped achieve a higher yield from Mg carbonation, which was 6.9% for the CA-added system compared to the non-CA system (0.9%). In the latter system, the variation of the Mg^2+^ concentration was virtually Δ*M* ∼ 0, and would probably lead to a high standard deviation, since the used Mg^2+^ quantification method is not highly sensitive at very low concentrations. It is possible that the carbonation in the absence of CA had not reached equilibrium and more time was needed. The presence of calcite has been proven to promote Mg carbonation in Mg-bearing silicates,^56^ which could also have happened here with the olivine. Furthermore, mineral carbonation benefits from an increased ionic strength of the solution, which was slightly higher here in the system containing the enzyme. Yet, no additives (salts) to enhance the solution ionic strength were added in the reactions performed in this study. The direct effect of CA in improving the CO_2_ storage set of reactions could be debated, but there was a clear indirect effect. First, CA boosted the CO_2_-capture reaction, producing higher amounts of bicarbonate and H^+^ protons, which further boosted the olivine dissolution step, causing an increased availability of Mg^2+^ ions. At the end of the carbonation reaction, 43.4 ± 3.0% of the available bicarbonate was consumed from the supernatant for the no-CA-added system and 61.4 ± 6.0% by the CA-added system, which could be explained by the formation of insoluble carbonate salts and the degassing that happened during the heating.

As mentioned in the previous section, studies showed that at conditions of mild temperature and pressure, and low or zero concentration of added salts, mineral carbonation was verified to be controlled by the ions diffusion through a Si-rich layer formed around the olivine core,^57^ which limits the H^+^ attack on the unreacted mineral, impairing the mineral dissolution, and consequently leading to a low carbonation yield, as also observed in this study. In addition, the use of bovine CA (BCA) for the mineral carbonation of Wollastonite was shown to bring a faster pH increase in the system, and therefore higher carbonation rates when compared to the control reaction.^58^ However, since the activity of the CA used in this system was not measured during the mineral dissolution and carbonation, it could not be confirmed that the increased carbonation was related with the presence of the enzyme.

Other studies using Ca/Mg-silicates for CCS have reported carbonation yields as high as 100%;^59^ however, they were generally performed under conditions such as high pressure, temperature, and CO_2_ concentration, which could play a role in improving the carbonation extent.^60,61^ The addition of salts (like NaHCO_3_) that can buffer the solutions was also suggested could keep the pH value controlled and increase the ionic strength to favor the carbonation,^62^ a strategy also adopted by some works to optimize the carbon sequestration.^63,64^ TGA of olivine before and after the CO_2_-storage reactions was performed. A maximum difference of 0.9% in total mass loss was observed between them. Pure olivine contains CaCO_3_, while small amounts of MgCO_3_ (and CaCO_3_) were possibly formed during the carbonation reaction. The first mass loss happened between 30–120 °C (5.0% of total mass loss), which was due to the moisture content in the analyzed samples. Between 350–600 °C, a notable proportion of the total mass variation was observed, ranging from 37.5%–41.3% for the analyzed samples, which could be attributed to the decomposition of other silica-based materials, such as serpentine.^65^ About 50% of the total mass loss occurred between 600–900 °C, linked to the thermal decomposition of CaCO_3_. It is important to note that between 300–350 °C, a slight mass loss of 0.9% between the unreacted and reacted olivine was noted, which could indicate the formation of nesquehonite (MgCO_3_·3H_2_O).^66^ Based on the quantification of the carbonation yield during our experiment, it is expected that the amount of MgCO_3_ formed during the mineral carbonation would be very small, which can explain why no significant mass loss in the full range of MgCO_3_ thermal decomposition was found. Besides, the XRD analysis of the olivine showed no detectable differences between before and after the reaction. The XRD analysis results and TGA curves are presented in Fig. S1 and S2 in the ESI.†Table 6 list some distinct studies on the mineral carbonation of silicate materials.

**Table 6:** Reported carbonation studies using different rock minerals

Main mineral	Concentration (g L^−1^)	Temperature (°C)	Pressure	Volume of reaction (L)	CO_2_ intake	% leaching	Carbonation yield and CO_2_ fixed	Residence time	Ref.
Serpentine	150	Room temperature	8 atm	∼6.2	14–18% CO_2_	17.3 (Mg)	17.9%, 0.215 g CO_2_ per g solid	6 h	48
Olivine	100	185	6.5 MPa	0.045	18.2% CO_2_	34 (Mg)	74.8%, N.Q.	Up to 6 h	63
Serpentine/lizardite	50–150	22	10.5 atm	0.3 (reactor size)	18.2% CO_2_	N.Q.	30%, 0.55 g CO_2_ per g solid	2.25 h	61
Lizardite	150	100–150	20–150	∼0.3	100% CO_2_	N.Q.	30%,^a^ N.Q.	Up to 6 h	64
Lizardite	150	Room temperature	0.4–1.6 bar	∼5.0	14–18% CO_2_	∼15.8%	33.3%, 0.08 g CO_2_ per g solid	6 h	60
Olivine	∼200	Up to 190	40–100 bar	0.01	100% CO_2_	N.Q.	100%, N.Q.	Up to 6 h	59
Rich Mg-bearing silicates	100	185	20.7–38.6 bar	0.6 (reactor size)	100% CO_2_	N.Q.	71%, N.Q.	5 h	57
Olivine	150	Room temperature (mineral dissolution) – 35–40 °C (carbonation)	3 bar (dissolution) – ambient pressure (carbonation)	0.17	1 g per L HCO_3_^−^	0.4% (Mg)	6.9%, N.Q.	5 days (mineral dissolution) + 12 h (carbonation)	This study (not optimized)
Wollastonite	2	Room temperature	2 bar	2	100% CO_2_	N.Q.	N.Q., 0.14 g CO_2_ per g solid	22 days	68

a MgCO_3_ yield.

As possibilities for improvement, we suggest longer residence times and/or higher temperature as means to increase the yield of carbonation, since the latter could lead to an enhancement in the kinetics of both the olivine dissolution and the carbonation.^67^ Furthermore, the carbonation experiments were conducted in non-pressurized vessels, which are more likely to be affected by degassing, decreasing the amount of CO_2_ (bicarbonates) over time, especially under heating, and therefore the utilization of reactors under pressure are recommended. Increasing the supernatant's bicarbonate concentration is another important parameter to be tested in olivine dissolution, which should be a topic of further studies to optimize the conditions for CO_2_ capture.

## Conclusions

Investigation of the routes to valorize alkaline residues is valuable, and their utilization for CCS tackles both CO_2_ mitigation and waste repurposing. Using CaCO_3_-rich residues generated in the Kraft process, a route for CCS was proposed in the present study and showed encouraging results. The utilization of CA improved significantly up to 5-fold the CO_2_ capture and almost 2-fold the mineral dissolution of olivine, when compared to a reaction without addition of the enzyme.

Some limitations on the mineral dissolution and carbonation were observed, probably because of the formation of a passivation rich Si-layer around the core of unreacted olivine impeding H^+^ from attacking the mineral, decreasing its dissolution rate. In addition, the formation of carbonates during the olivine dissolution step with the pH increase over time can strongly inhibit the mineral dissolution rate, leading to a low concentration of Mg^2+^ leached. The carbonation extent of Mg^2+^ ions was 7.1-fold higher using bicarbonate from a CA-added capture/dissolution reaction compared to the no-CA-added case, which could potentially be improved in future studies. A carbonation yield of 6.9% was achieved. As recommendations, the utilization of pressurized vessels for mitigating the effects of degassing, longer residences times, and more concentrated bicarbonate solutions could lead to a higher availability of the leached metals, accelerating the carbonation, and so should be considered in future studies. Nevertheless, we successfully demonstrated a proof-of-concept CCS strategy for the paper and pulp industry, which could be achieved even at relatively low pressures (3 bar). Tests at higher pressures will be part of future work in order to simulate geological conditions during the injection of bicarbonate in the bedrock (>100 bar), which will certainly benefit the carbonation yields.

## Conflicts of interest

There are no conflicts to declare.

## Supplementary Material

RA-014-D3RA06927C-s001
