# Supplementary material for: Enzyme-accelerated CO2 capture and storage (CCS) using paper and pulp residues as co-sequestrating agents
Source: RSC Adv. 2024 Feb 20;14(9):6443–61. doi: 10.1039/d3ra06927c (PMC10878411; doi:10.1039/d3ra06927c)
Supplement: RA-014-D3RA06927C-s001 [file RA-014-D3RA06927C-s001.pdf]

Electronic Supplementary Material (ESI) for RSC Advances.
This journal is © The Royal Society of Chemistry 2024

Enzyme-accelerated CO_2 capture and storage (CCS) using paper and pulp residues as co-sequestrating agents

Ayanne De Oliveira Maciel, Paul Christakopoulos, Ulrika Rova, Io Antonopoulou

Biochemical Process Engineering, Division of Chemical Engineering, Department of Civil, Environmental and Natural Resources Engineering, Luleå University of Technology, SE-97187 Luleå, Sweden

Supporting information

1. Calculations for CO_2 capture step

This study considered the main paths for the bicarbonate formation: the CO_2 hydration, and the mineral dissolution of $CaCO_3$, $Mg(OH)_2$ and $Ca(OH)_2$. The possible influence of other materials as CO_2 co-sequestrating agents was not considered in this study scope. The carbon balance was performed according to the presence of the materials as presented in Table S1.

Table S1: Reactions for CO_2 capture for each evaluated residue

	CO_2 hydration	$CaCO_3$ dissolution	$Mg(OH)_2$ dissolution	$Ca(OH)_2$ dissolution
R1	Yes	Yes	No	No
R2	Yes	Yes	Yes	No
R3	Yes	Yes	No	No
R4	Yes	Yes	No	Yes

Reactions considered to contribute to CO_2 capture:

CO_2 hydration:

$$CO_{2(g)} + H_2O \leftrightarrow HCO_3^- + H^+ \qquad \text{eq. S1}$$

$CaCO_3$ dissolution:

$$CaCO_3 + H^+ \leftrightarrow Ca^{2+} + HCO_3^- \qquad \text{eq. S2}$$

$Mg(OH_2)$ dissolution:

$$Mg(OH)_2 + 2H^+ + 2CO_{2(g)} \leftrightarrow Mg^{2+} + 2HCO_3^- \qquad \text{eq. S3}$$

$Ca(OH)_2$ dissolution or Ca^{+2} precipitation (For pH>8, bicarbonate converts to carbonate that precipitates to $CaCO_3$):

$$C\alpha(OH)_2 + 2H^+ + 2CO_{2(g)} \leftrightarrow Ca^{2+} + 2HCO_3^-$$

$$HCO_3^- \leftrightarrow CO_3^- + H^+$$

$$Ca^{2+} + CO_3^{2-} \leftrightarrow CaCO_{3(s)} \qquad \text{eq. S4}$$

The total molar concentration of bicarbonate (HCO_3^-) in the solution at end point:

$$\left[HCO^-_{3,mineralization\ method}\right] = \left[HCO^-_{3,\ CO_2\ hydration}\right] + \left[HCO^-_{3,\ CaCO_{3,dissolution}}\right] + \left[HCO^-_{3,\ Mg(OH)_{2,dissolution}}\right]$$

From eq. S2:

$$\left[HCO^-_{3,\ CaCO_{3,dissolution}}\right] = \Delta\,[Ca^{2+}] = [Ca_t^{2+}] - [Ca_{t=0}^{2+}] > 0 \qquad \text{eq. S5}$$

From eq. S3:

$$\left[HCO_{3,\ Mg(OH)_{2,dissolution}}^{-}\right] = \frac{\Delta[Mg^{2+}]}{2} = \frac{[Mg_t^{2+}]-[Mg_{t=0}^{2+}]}{2}$$ (From eq. S3) eq. S6

Thus:

$$\left[HCO_{3,\ CO_2\ hydration}^{-}\right] = \left[HCO_{3,mineralization\ method}^{-}\right] - \left[HCO_{3,\ CaCO_{3,dissolution}}^{-}\right] - \left[HCO_{3,\ Mg(OH)_{2,dissolution}}^{-}\right]$$ eq. S7

From eq. S4:

$$\left[HCO_{3,\ Ca(OH)_{2,dissolution}}^{-}\right] = \left[CO_{3,\ Ca(OH)_{2,dissolution}}^{2-}\right] = \frac{\Delta[Ca^{2+}]}{2} = \frac{[Ca_t^{2+}]-[Ca_{t=0}^{2+}]}{2} < 0$$ eq. S8

Finally, the total molar concentration of CO_2 capture in bicarbonate equivalent is:

$$\left[HCO_{3,\ total}^{-}\right] = \left[HCO_{3,mineralization\ method}^{-}\right] + \left[HCO_{3,\ Ca(OH)_{2,dissolution}}^{-}\right] = \left[CO_{2,\ total}\right]$$ eq. S9

The concentration of CO_2 captured (g/L):

$$C = \frac{[HCO_3^-]\ \cdot 44}{61}$$

2. Calculations for CO_2 storage step

Reactions considered to contribute to CO_2 storage:

Mineral dissolution:

$$Mg_2SiO_4\ +\ 4H^+\ \leftrightarrow\ 2Mg^{2+}\ +H_4SiO_4$$ eq.10

Mineral carbonation:

$$Mg^{2+}\ +\ CO_3^{2-}\ \leftrightarrow\ MgCO_{3\ (s)}$$ eq.11

The solution used as source of bicarbonate (HCO_3^-) from the CO_2 capture step contains Mg^{2+} and Ca^{2+}.

The concentration of Mg^{+2} ions leached during the olivine dissolution is calculated as:

$$\left[Mg_{Olivine\ dissolution}^{2+}\right] = \Delta[Mg^{2+}] = [Mg_t^{2+}] - [Mg_{t=0}^{2+}] = [Mg_t^{2+}] - \left[Mg_{CO2\ capture}^{2+}\right]$$ eq. S10

The concentration of Ca^{+2} ions was proven to decrease over time during olivine dissolution, forming insoluble salts.

The yield of mineral carbonation can be calculated considering only formation of $MgCO_3$, as Mg is mainly the element that derives from the olivine mineral during dissolution. Mg^{+2} is the limiting reactant, according to tested conditions: 1 g/L HCO_3^-= 0.016 mol/L and max 154 mg/L Mg^{+2}=0.00625 mol/L. From eq. S11:

$$Carbonation\ yield\% = -\frac{[Mg_t^{2+}]-[Mg_{t=0}^{2+}]}{[Mg_{t=0}^{2+}]} \cdot 100 = -\frac{[Mg_t^{2+}]-[Mg_{olivine\ dissolution}^{2+}]}{[Mg_{olivine\ dissolution}^{2+}]} \cdot 100$$ eq. S11

3. Mass balance XRD and XRF

For estimating the concentration of the main phases in the studied residues a mass balance was performed comparing the data obtained from XRF and XRD analysis. The first was utilized to calculate the elements concentration and the latter the main phases found in the residue. Some simplifications were done during the mass balance, which are:

1) Elements found with concentration lower than 0.3% (XRF) were not considered in mass balance of the phase's concentration determination.
2) The concentration of each phase was tested to "close" the element mass balance:
    a. $\%Total\ Element_{XRF} = \sum_i^n \%Element_{phase_{i,XRD}} = \sum_i^n (\%Phase_{i,XRD} \times MM_{element}/MM_{phase,i})$.
    b. The elements not considered in the mass balance (%w < 0.3) were assumed to belong to other phases .
    c. $\sum_i^n \%Phase_{i,XRD}$ ~ 100.00% ($(\sum_i^n \%Phase_{i,XRD} - 100\%)/100\% < 1.0\%$).

Where:

$\%Element_{XRF}$ is the percentage (in %w) measured in XRF analysis.

$\%Phase_{i,XRD}$ is the attributed percentage (in %w) of the phase.

In table S2, an example of the how the final mass balance of a residue would look like.

Table S2: Example of mass balance for a reference material.

**Phase/Element**	**% phase**	**Ca**	**Mg**	**C**	**O**	**Al**	**H**	**Fe**
**$CaCO_3$ (Calcite)**	$\%Phase_{CaCO_3,XRD}$	$\%Element_{phase_{CaCO_3,XRD}}$	…				…	
**$Mg(OH)_2$**	…	…						
**CaO**								
**Kieserite ($MgSO_5H_2$)**								
**Almandine ($Fe_3Al_2Si_3O_{12}$)**								
**Other phases**	$\%Phase_{other\ phases}$	…						
**Total**	100	$\%Ca_{XRF}$	…					$\%Fe_{XRF}$

4. XRD analysis – Olivine before and after the CO_2 storage reaction

Olivine prior and after the dissolution/mineral carbonation (in presence of bicarbonate from no CA-added CO_2 capture reaction and bicarbonate from a CA-added CO_2 capture reaction) were analyzed. Results did not point significant changes between the samples before and after reaction.

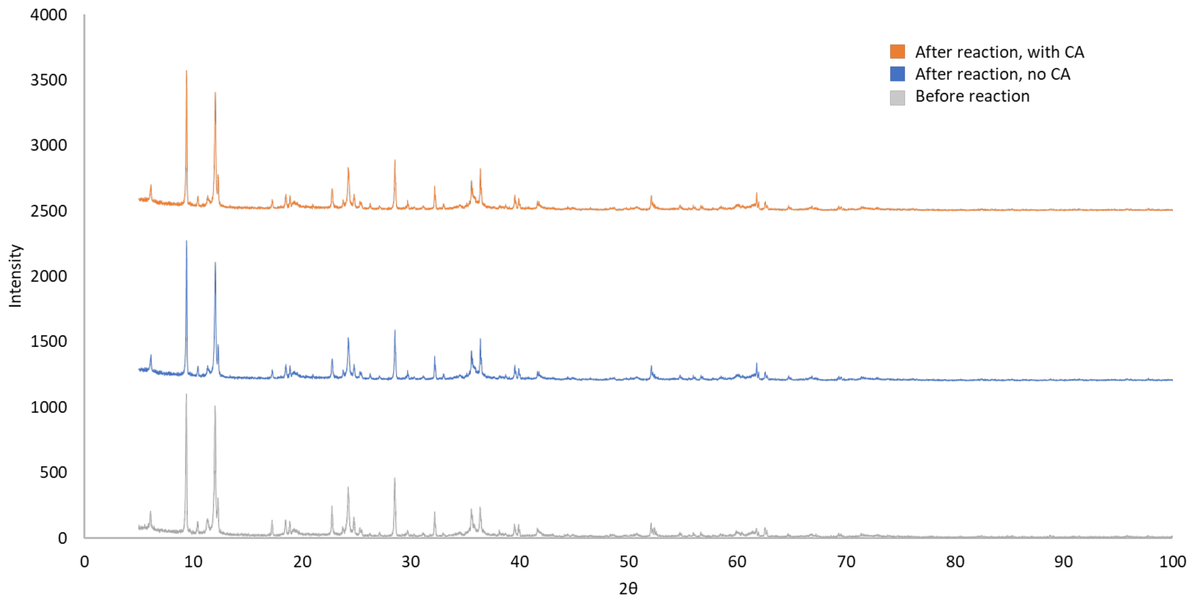

Figure S1: XRD spectra of olivine - before and after reaction

5. TGA – Olivine before and after reaction the CO_2 storage reaction

TGA analysis showed that the olivine after reaction (using bicarbonate from no CA-added CO_2 capture reaction and using bicarbonate from a CA-added CO_2 capture reaction) presented higher mass loss when compared to the unreacted mineral. Some moisture was identified, corresponding to the mass loss between 30-130ºC. Between 500-900ºC the mass loss for all materials was 74.5-75.4%, indicating the presence of $CaCO_3$. Between 300-350ºC, both no- and CA added systems presented a slight difference (around 0.9%) in mass decrease when compared to non-reacted olivine – which could be due the formation of nesquehonite ($MgCO_3.3H_2O$), as showed in S4.

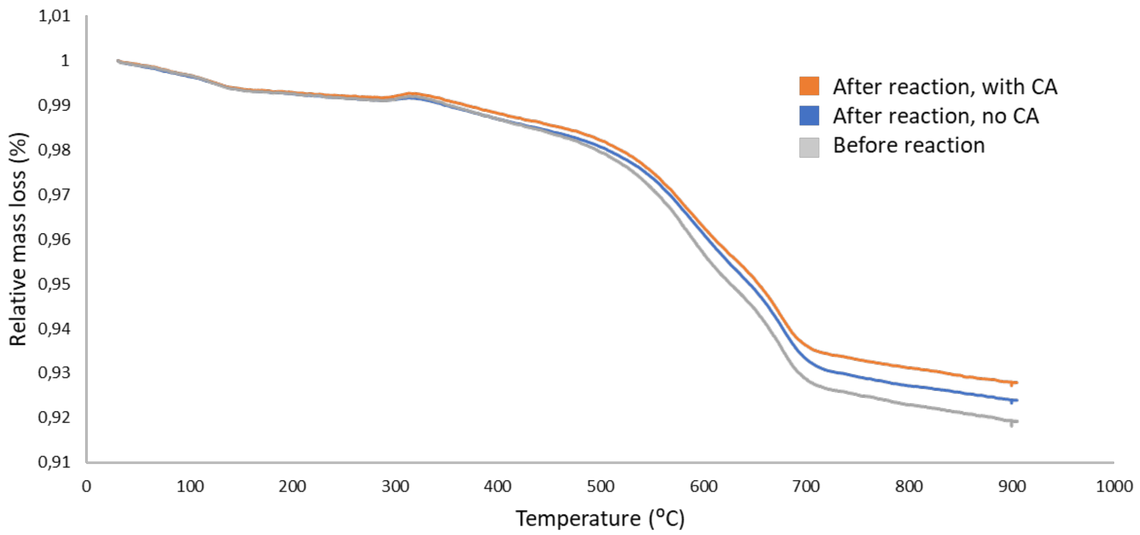

Figure S2: TGA of olivine before and after reaction
